# Late Presentation of Acute Coronary Syndrome Complicated by Ventricular Septal Rupture

**DOI:** 10.7759/cureus.43427

**Published:** 2023-08-13

**Authors:** Daniel Sherlock, Andrii Labchuk, Umar Hussain, Muhammad A Khan, Piotr Wlodkowski, Nishant Patel

**Affiliations:** 1 Internal Medicine, Advocate Lutheran General Hospital, Park Ridge, USA; 2 Cardiology, Advocate Lutheran General Hospital, Park Ridge, USA

**Keywords:** post-myocardial infarction vsr, acute coronary syndrome (acs), ventricular septal defect (vsd), cardiology research, stemi

## Abstract

Acquired ventricular septal rupture (VSR) is a rare but potentially fatal complication of late-presenting myocardial infarction (MI). In the era of revascularization and reperfusion therapy, the incidence of VSR has significantly decreased. Ruptures occur predominantly in patients with late-presenting ST elevation MI. Patients may present with a wide variety of symptoms ranging from chest pain and mild hemodynamic instability to profound cardiogenic shock. Inotropes, vasopressors, and mechanical support with intra-aortic balloon pumps and extracorporeal membrane oxygenation can be used to bridge patients to surgery. Despite treatment with ventricular septal repair, postsurgical mortality remains high. There is a wide variety of complications that can occur in the postoperative period. A multidisciplinary approach is vital in these patients who develop VSR. Improving awareness among healthcare professionals regarding the symptoms of acute coronary syndrome can hopefully help prevent delayed presentation of patients to healthcare facilities.

## Introduction

Ventricular septal rupture (VSR) has become much rarer with the advent of aggressive reperfusion strategies in patients with myocardial infarction (MI). However, in the cases that do present, mortality is very high. These cases usually occur within a week of MI and present with hemodynamic instability. Prognosis is associated with the size of the rupture, with smaller ruptures having better outcomes [1]. Management of VSRs is surgical, with the definitive treatment being the closure of the defect. In this report, we would like to present a case of a late-presenting MI complicated by VSR. Clinical features of VSR can range from mild symptoms to profound cardiogenic shock [1,2]. Thus, early identification and treatment can be lifesaving. Despite successful repair of VSR, postsurgical morbidity and mortality remain high.

## Case presentation

A 52-year-old male, with a past medical history of hypertension, hyperlipidemia, and ulcerative colitis, presented to the emergency department (ED) via emergency medical services (EMS) for evaluation of substernal chest pain. The patient had been complaining of chest pressure and shortness of breath for the past week. His symptoms progressively worsened throughout the course of the week until they became unbearable. An electrocardiogram (ECG) obtained by the EMS in the field showed ST segment elevations in the inferior leads. The patient received 324 mg of aspirin and was transported to the ED. Upon arrival at the ED, the patient was hypotensive, tachycardic, and complaining of persistent chest pain (Table 1).

**Table 1:** Vital Signs on Arrival

Heart rate	Respiratory rate	Blood pressure	SpO2	EtCO2	Temperature
131 bpm	32 breaths/minute	137/67 mmHg	92%	18 mmHg	97.3 F

Labs were notable for creatinine of 1.34 mg/dL, aspartate aminotransferase of 57 U/L, alanine aminotransferase of 125 U/L, troponin I of 5143 ng/mL, PT/PTT/INR within normal limits, NT-proBNP of 845 pg/mL, and white blood count of 13.1 K/mcL.

**Figure 1:**
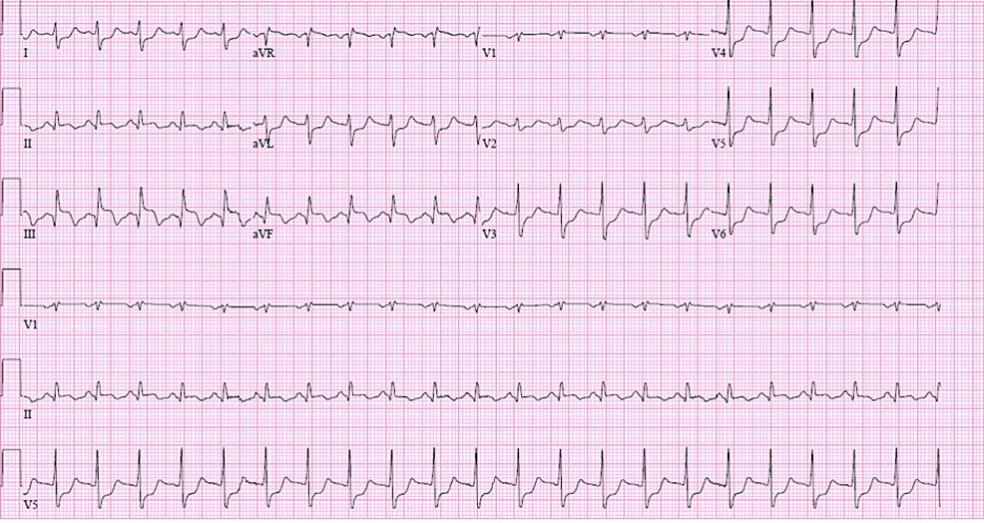
ECG on Presentation

ECG on presentation showed sinus tachycardia with an inferior injury pattern and lateral ST depression concerning an acute MI (Figure 1). 

The patient received a heparin bolus and 80 mg of atorvastatin in the emergency department and was taken emergently to the cardiac catheterization lab. Coronary angiography showed 100% occlusion of the proximal right coronary artery (RCA), 50% distal stenosis of the left main coronary artery, diffuse long 70-80% stenosis of the left anterior descending (LAD), and diffuse 70% mid-stenosis of the circumflex branch of the left coronary (LCx) artery. A 2.5x22 mm Orsiro drug-eluting stent was placed with residual thrombolysis in MI (TIMI) three flow. During the procedure, the patient developed a worsening shock requiring the initiation of a norepinephrine drip. A left ventriculogram was performed which showed a hyperdynamic left ventricle (LV) with a left-to-right shunt and large ventricular septal defect (Video 1). 

**Video 1:** Left Ventriculogram Showing Ventricular Septal Defect (VSD) Left-to-right shunting of contrast seen on left ventriculography suggestive of a large VSD. Confirmed later with TTE

Qp/Qs was calculated to be 2.65 indicating a significant left-to-right shunt. Right heart catheterization was performed with findings determined using the Fick method (Table 2). 

**Table 2:** Right Heart Catheterization Findings

Right atrial pressure	Right ventricular pressure	Mean PA pressure	Pulmonary capillary wedge pressure	Cardiac output	Cardiac index
30 mmHg	64/19 mmHg	43 mmHg	33 mmHg	3.23 L/min	1.5 L/min/m²

**Figure 2:**
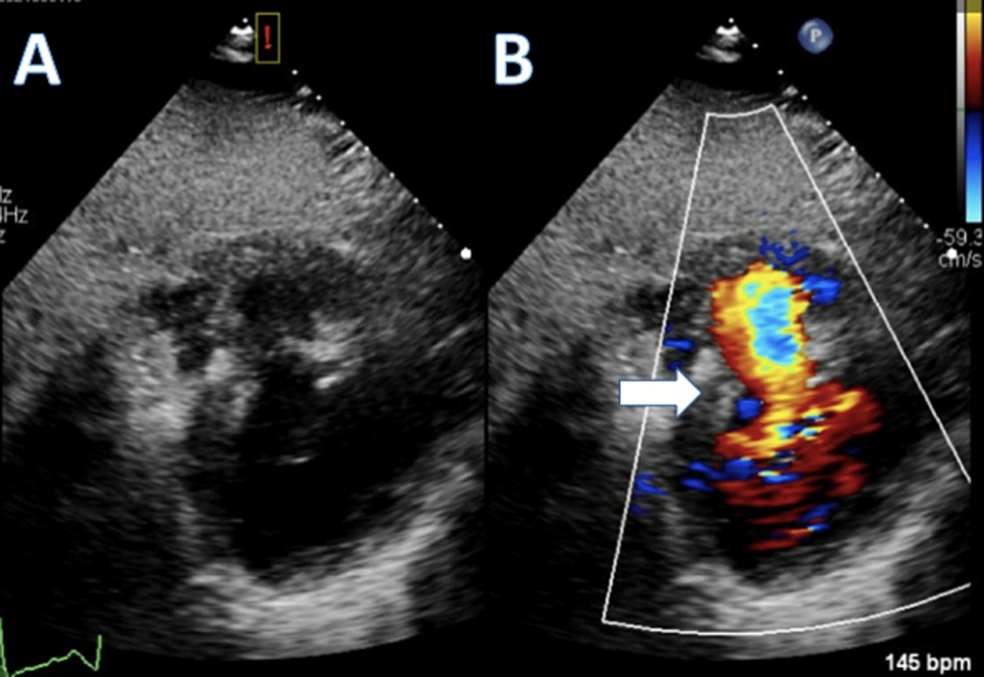
Transthoracic Echocardiogram Showing Ventricular Septal Defect A) Parasternal short axis view showing the right ventricle superior to the left ventricle; a defect is seen in the intraventricular septum. B) Color Doppler showing aberrant blood flow between the right and left ventricles through a defect in the septal wall (white arrow)

Aortic saturation was 95%, and mixed venous saturation was 78%, with right atrial/inferior vena cava saturation of 41%, indicating a significant step up in oxygenation. Due to a significant left-to-right shunt and a large ventricular septal defect, an intra-aortic balloon pump was placed for mechanical support and afterload reduction to reduce the shunt. A Swan-Ganz catheter was left in place for hemodynamic monitoring. The patient received one dose of IV furosemide, was loaded with ticagrelor, and was transferred to the medical cardiac intensive care unit (MCICU). Upon arrival, the patient was noted to be in significant respiratory distress and was started on a high-flow nasal cannula. However, the patient soon had to be intubated due to worsening respiratory distress. Transthoracic echocardiogram (TTE) demonstrated a large ventricular septal defect, hyperdynamic systolic function of the left ventricle, and an 80% left ventricular ejection fraction (Figure 2). A small, free-flowing pericardial effusion was also identified.

The patient was started on a dobutamine drip for inotropic support, and cardiovascular surgery was consulted to evaluate the patient for extracorporeal membrane oxygenation (ECMO). After a long multidisciplinary discussion, the decision was made to cannulate the patient for peripheral VA-ECMO via the left femoral artery and right femoral vein. After successful cannulation, the patient was transferred to another hospital for a higher level of care. Unfortunately, the patient’s hospital course was complicated by recurrent cardiac tamponade requiring pericardial window creation. He ultimately underwent VSR repair with a Bard polytetrafluoroethylene (PTFE) patch (Bard Peripheral Vascular, Inc., Arizona) and a three-vessel coronary artery bypass graft (CABG) on day 11 of his initial presentation. The postsurgical course was complicated by acute kidney failure that required continuous renal replacement therapy, ischemic hepatitis, and gastrointestinal bleeding that required multiple blood transfusions. Subsequently, the patient developed necrotizing pneumonia secondary to Klebsiella, Proteus, and blastomycosis. His course was also complicated by an ischemic stroke, which was thought to be an embolic stroke of undetermined source (ESUS). Unfortunately, the patient ultimately expired after the family decided to withdraw care due to multiple organ system failure.

## Discussion

VSR, free wall rupture, and acute mitral regurgitation are severe and potentially lethal mechanical complications of MI. They have similar underlying pathophysiology: rupture or tearing of necrotic myocardium due to MI [2]. We will discuss the risk factors, management, and complications of VSR. 

VSR is relatively rare, but the most common mechanical complication related to MIs. Incidence has decreased with the widespread use of reperfusion therapy. It is more common in patients with STEMI compared to non-STEMI (NSTEMI) likely due to the larger, more significant area of damage seen in STEMIs. VSR occurs in 0.21% of the STEMI and 0.04% of the NSTEMI populations. Despite surgical repair, in-hospital mortality remains high with an in-hospital mortality rate of 42.4% after STEMI and 18% after NSTEMI [3]. 

A transmural MI in one of the following arteries can lead to VSR: 

The left anterior descending coronary artery, which supplies most of the anterior portion of the interventricular septum and can lead to apical VSR. 

The dominant right coronary artery, which supplies the inferior portion of the interventricular septum and can lead to a basal VSR. 

Coagulation necrosis is the most common pathological finding in VSR. This process usually takes three to five days, but it can also occur within 24 hours of MI. Coagulation necrosis develops in the area of the MI due to the denaturation of proteins secondary to the lack of oxygen from the loss of blood supply. After developing the shunt between the right and left ventricle due to VSR, oxygenated blood shunts from the high-pressure left ventricle to the low-pressure right ventricle [1]. 

Prior to the wide use of reperfusion therapy, the risk factors for VSR were female sex, hypertension, advanced age, and absence of a previous history of MI or coronary artery disease (CAD). CAD and previous MI were noted to be somehow cardioprotective in terms of developing VSR due to the formation of collateral coronary vessels [4]. Patients who present with acute coronary syndrome (ACS) complicated by VSR usually rapidly progress to cardiogenic shock. Echocardiogram is the gold standard for the diagnosis of VSR and helps to differentiate it from other mechanical complications with a sensitivity and specificity of 100%. Echocardiography is a helpful tool to estimate the rupture and severity of the shunt. Color Doppler echocardiography is also useful in the evaluation of the anatomical size of a rupture [5]. 

Patients who develop a rupture of the intraventricular septum may present with a wide variety of symptoms, from chest pain and mild hemodynamic compromise to severe cardiogenic shock with hypotension and tachycardia. Biventricular heart failure may also be present. A new, harsh, loud, and holosystolic murmur is nearly always present and is heard best at the lower left and right sternal border, but it can be heard across the precordium [6].

The definitive treatment of VSR is surgical repair [7]. VSR repair should not be delayed as most patients will not survive the delay due to progressive heart failure and/or multiorgan failure and infection [8,9]. As a bridge to surgery in patients with cardiogenic shock, the patient should be stabilized using inotropic agents and vasopressors. These agents may also decrease the left-to-right shunt associated with the VSR. Inotropic agents may be required in patients with low cardiac output. Vasopressors can increase afterload and may actually worsen the left-to-right shunt. An intra-aortic balloon pump (IABP) is vital for temporary hemodynamic support. This device lowers afterload and decreases the shunt while also facilitating coronary perfusion [10]. Surgical repair of a VSR carries a relatively high mortality risk and suboptimal results with a postoperative residual shunt in up to 20% of cases [11]. In a study on early outcomes of postinfarction VSR, the most common causes of death were attributed to low cardiac output syndrome, a syndrome where inadequate systolic function results in hypoperfusion and tissue hypoxia, and multiorgan failure [12]. Given the poor results of surgery, the technique of percutaneous VSR device closure has been developed as a less invasive approach and has a high procedural success rate. Percutaneous VSR closure offers an alternative to surgery; however, mortality of postinfarction VSD remains high, particularly in patients with cardiogenic shock [13]. 

Following surgery, postoperative complications are common including cerebral vascular accidents, acute kidney injury (AKI) requiring renal replacement therapy, pneumonia, and heart block. Postoperative AKI is a serious, multifactorial condition that carries a high mortality risk. However, the most common cause of postoperative AKI is acute tubular necrosis due to hypotension or hypovolemia. CABG during VSR repair reduces both early and late mortality when compared with patients who did not have CABG [14]. When medical management and IABP fail to medically stabilize patients in cardiogenic shock, ECMO should be considered to stabilize and allow time to develop a surgical plan for VSR repair [15]. 

## Conclusions

VSR, free wall rupture, and papillary muscle rupture are well-known mechanical complications of MI. VSR, although relatively rare, is the most common mechanical complication related to late-presenting MI. Due to the more widespread use of reperfusion and revascularization therapy, the incidence of VSR has significantly decreased. However, VSR remains a life-threatening complication of MI with a high mortality rate despite surgical intervention. It is important to identify VSR early and start appropriate treatments to bridge the patient to surgery. IABP and VA-ECMO remain important tools for stabilizing patients until surgery. Postoperative complications after VSR repair include but are not limited to cerebral vascular accidents, acute renal failure requiring renal replacement therapy, pneumonia, and heart block. Our case provides a reminder of the significance of mechanical complications of MI and their associated high mortality. 
